# Supplementary material for: Performance of aging consumers in an e-commerce product choice task: The role of working memory and decision strategies
Source: PLoS One. 2024 Jun 21;19(6):e0303806. doi: 10.1371/journal.pone.0303806 (PMC11192353; doi:10.1371/journal.pone.0303806)
Supplement: S1 File — (DOCX) [file pone.0303806.s001.docx]

**S1. Decision Diﬃculty x Phase interaction**

Decision Diﬃculty x Phase interaction, F (6, 876) = 9.30, MSE = .07, p < .001, *η*_p_^2^ = .060 ( S1 Fig).


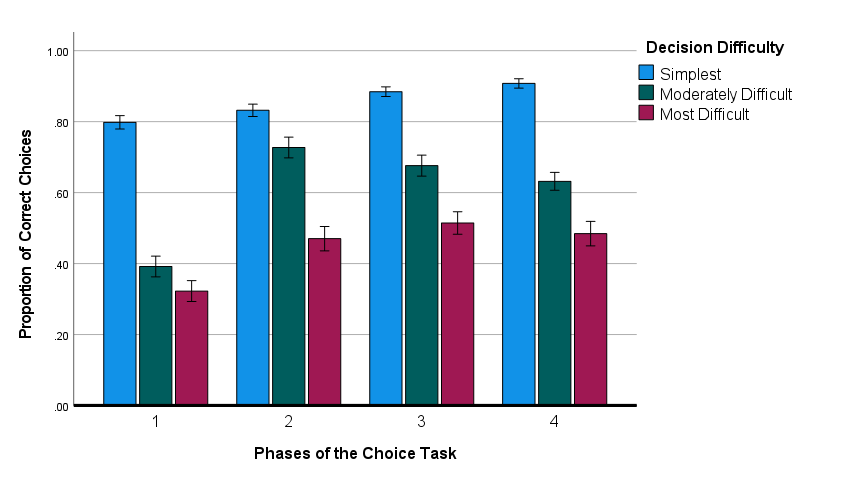


**Fig.** **S1. Proportion of correct choices in the multi-attribute decision task (Part I with feedback) as a function of phases of the task and decision difficulty.** **Error bars represent standard errors.**

The increase in performance in phase 2 in comparison to that in phase 1 and further lack of improvement were also characteristic of the moderately and most-difficult tasks. However, (S1 Fig), for the simplest decision task, there was a linear increasing trend across all four phases (p < .001).

**S2. Decision Diﬃculty x Part of Decision Task interaction**

There was a significant increase in performance in part II compared to part I (p < .001) for the simplest and moderately difficult decision tasks and a lack of improvement for the most-difficult tasks.


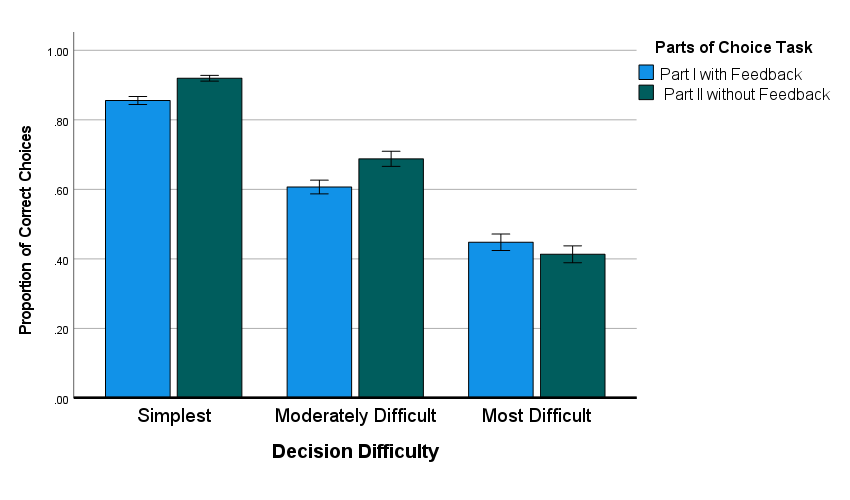


**Fig. S2. Decision Diﬃculty x Part of Decision Task, F (2,292) = 13.59, MSE = .02, p < .001, *η*_p_^2^ = .085. Error bars represent standard errors.**

**S3. Correlation of Independent Variables Separately for Three Age Groups.**

**Table S3. Correlation Table of Independent Variables Separately for Three Age Groups.**

| Age group | Variable | 1 | 2 | 3 | 4 | 5 | 6 |
| --- | --- | --- | --- | --- | --- | --- | --- |
| Younger adults | 1. Acc_Dec | - |  |  |  |  |  |
|  | 2. Tm_Dec | *.*51^∗∗^ | - |  |  |  |  |
|  | 3. VWM | .50^∗∗^ | .27 | - |  |  |  |
|  | 4. Helplessness | -*.*33^∗^ | -.38^∗∗^ | *-.*04 | - |  |  |
|  | 5. S Numeracy | .31^∗^ | *.*10 | .14 | -.03 | - |  |
|  | 6. NFC_4subs | -.18 | -.23 | -.10 | .15 | *-.*14 | - |
| Middle-aged adults | 1. Acc_Dec | - |  |  |  |  |  |
|  | 2. Tm_Dec | *.*03 | - |  |  |  |  |
|  | 3. VWM | .41^∗∗^ | -.31^∗^ | - |  |  |  |
|  | 4. Helplessness | -*.*31^∗^ | .14 | *-.*32^∗^ | - |  |  |
|  | 5. S Numeracy | .30^∗^ | *.*00 | .35^∗^ | -.03 | - |  |
|  | 6. NFC_4subs | -.11 | .07 | -.14 | .10 | .04 | - |
| Older adults | 1. Acc_Dec | - |  |  |  |  |  |
|  | 2. Tm_Dec | *.*11 | - |  |  |  |  |
|  | 3. VWM | .32^∗^ | -.14 | - |  |  |  |
|  | 4. Helplessness | .05 | -.08 | *-.*16 | - |  |  |
|  | 5. S Numeracy | .20 | -.17 | .16 | -.15 | - |  |
|  | 6. NFC_4subs | .04 | -.03 | -.15 | .00 | .22 | - |

*Note:* Acc_Dec = Accuracy of Decision Task; Tm_Dec = Time of Decision Task; VWM= Visual Working Memory Task; Helplessness = Scale of Helplessness of Contracting an Infectious Disease; S Numeracy = Subjective Numeracy Scale; NFC_4subs = 4 subscales of the Need for Cognitive Closure Short Scale. *p *<* .05, **p *<* .01

**S4. Description of the Features.**

For the feature ‘energy label’, there were four values: A+++ (best value), A++, A+, and A (worst value). For the feature ‘water usage’, there were six values: 30 (best value), 40, 45, 50, 55, and 65 (worst value). For the feature ‘noise level’, there were six values: 40 (best value), 45, 50, 60, 65, and 70 (worst value). For the feature ‘quick wash’, there were two options: yes (better option) or no (worse option). For the feature ‘capacity’, there were five values: 12 (best value), 10, 8, 6, and 4 (worst value). For the feature ‘maximum spin speed’, there were four values: 1600 (best value), 1400, 1200, and 1000 (worst value).

**S5. Description of the Quality and Delta Parameters.**

We have assumed that e-commerce consumers select products within a certain price range for comparison. We have decided to consider two scenarios: a selection of high-end products and a selection of low-end products. To prepare the two scenarios, we introduced two parameters: quality (Q) and delta (D). All products in tasks where Q was high had parameter values characteristic of high-end products (e.g., noise level in range 50-40 dB), while products with low Q had the worse values characteristic of low-end products (e.g., noise level in range 70-60 dB). The D parameters specified the difference between better and worse parameter values within the same task. For example, for products with large D (large difference) and high Q, the participants compared washing machines generating 50 db with those generating 40 db, while for products with small D (small difference) and high Q, the parameters were 50 db and 45 db. By using the Q and D parameters, we were able to control for the distribution of attribute values in our product choice tasks and investigate the effect of attribute values on participants’ choices. Half the tasks in the experiment had a high Q, while half had a low Q (high-end and low-end product comparisons, respectively). Similarly, half the tasks had a low D, while half had a high D.
