# Supplementary material for: Performance of aging consumers in an e-commerce product choice task: The role of working memory and decision strategies
Source: PLoS One. 2024 Jun 21;19(6):e0303806. doi: 10.1371/journal.pone.0303806 (PMC11192353; doi:10.1371/journal.pone.0303806)
Supplement: Attachment — Submitted filename: Response to reviewers.docx [file pone.0303806.s002.docx]

# Response to Reviewer Comments for article PONE-D-23-38646

Dear Editor,

We are writing this letter in response to the comments of reviewers of the article:

PONE-D-23-38646

Performance of Aging Consumers in an E-commerce Product Choice Task: The Role of Working Memory and Decision Strategies.

Submitted to PLOS ONE.

We would like to thank all reviewers for their careful consideration of the article, and for their insightful comments. We have revised the article taking into account all comments of all reviewers. In particular, the new version of the article includes the following changes:=

1. Addition of references about research for purchase intent modeling using behavioral and eye-tracking data
2. Formatting of paper according to journal guidelines, in particular
   a. Headers
   b. Removing figures from the main article, and leaving correct figure captions and references to figures according to the journal guidelines
   c. References – adding DOI and PMID identifiers where possible
3. Checking figures’ compliance using Preflight Analysis and Conversion Engine (PACE) digital diagnostic tool. All submitted figures have been passed by PACE as compliant

Below please find detailed responses to reviewer’s comments:

*Reviewer #1: It sheds light on the relationship between the age of users and their decision-making mechanisms on e-commerce platforms. In this context, It can be said that it contributes to the extant literature. Therefore, I accept this paper.*

Thank you for your consideration and the positive review.

*Reviewer #2: Dear Authors*

*Thank you for your submission.*

*Here are some remarks and recommendations regarding your manuscript titled “Performance of Aging Consumers in an E-commerce Product Choice Task: The Role of Working Memory and Decision Strategies”.*

*The proposed paper reflects experiments on purchase intent prediction for elderly customers. Some individual characteristics including visual working memory were shown as reliable mediators of the relationship between age and decision accuracy.*

*The results are based on a simulation and not real e-commerce data.*

Our research bases on empirical experimentation with real users, not on simulation. The data used in the experiments for product comparison has been obtained from real e-commerce products (washing machines). This includes the features of the products, and the possible values of the features. However, the specific values for products in the comparison have been chosen by us, in order to control the difficulty of product comparison tasks, and also to account for comparison of high-end, or low-end features, as described in the article.

*Eye-tracking was not used in the study, which could have brought the results to the next level. All this should be thoroughly justified with view to updating the results in future papers based on real-world online shops and with more observation techniques.*

We thank you for this comment. We have added a paragraph in the “Conclusion and future work” section. Indeed, eye-tracking could be used in the investigation of how cognitive limitations affect product comparison, in particular for detecting cognitive fatigue, and to detect comparison strategies used by experiment participants.

*The literature review is far too scarce for such a wide topic and lot of important related studies from recent years have been omitted by the authors. I would expect a much wider review of studies on purchase intent modeling based on user tracking including AI analytics such as fuzzy sets and neural networks. Perhaps also some papers where eye-tracking was used too. This should be revised and elaborated on.*

Thank you for this comment. We have added a paragraph in the “Introduction” section about the use of behavioral and eye-tracking data for modeling and predicting purchase intent. We have also added several references on this topic.

*Other than that, the paper is timely and interesting, in particular with regard to the recent pandemic, where online content started to play an increasingly important role. The article in general is technically sound, and the conceptual research model convincing with some more work in studying the state-of-the-art and justifying the methods.*

*I look forward to the improvements.*

Thank you for your consideration and thorough review of our article.
